# Tear resistance of soft collagenous tissues

**DOI:** 10.1038/s41467-019-08723-y

**Published:** 2019-02-15

**Authors:** Kevin Bircher, Manuel Zündel, Marco Pensalfini, Alexander E. Ehret, Edoardo Mazza

**Affiliations:** 1 ETH Zurich, Institute for Mechanical Systems, 8092 Zurich, Switzerland; 2 Empa, Swiss Federal Laboratories for Materials Science and Technology, 8600 Dubendorf, Switzerland

**Keywords:** Biomaterials, Biomedical engineering, Bioinspired materials, Tissues

## Abstract

Fracture toughness characterizes the ability of a material to maintain a certain level of strength despite the presence of a macroscopic crack. Understanding this tolerance for defects in soft collagenous tissues (SCT) has high relevance for assessing the risks of fracture after cutting, perforation or suturing. Here we investigate the peculiar toughening mechanisms of SCT through dedicated experiments and multi-scale simulations, showing that classical concepts of fracture mechanics are inadequate to quantify and explain the high defect tolerance of these materials. Our results demonstrate that SCT strength is only modestly reduced by defects as large as several millimeters. This defect tolerance is achieved despite a very narrow process zone at the crack tip and even for a network of brittle fibrils. The fracture mechanics concept of tearing energy fails in predicting failure at such defects, and its magnitude is shown to depend on the chemical potential of the liquid environment.

## Abstract (web summary)

Defect tolerance is important in order to ensure the mechanical integrity of synthetic materials and biological tissues. Here the authors demonstrate that toughening mechanisms provide soft collagenous tissues with high defect tolerance, with important implications for biomedical applications.

## Introduction

Failure of materials is often associated with the presence of defects, either generated during manufacturing or originating from the growth and coalescence of microstructural faults. Reduction of tear resistance as a consequence of defects in soft biological tissues is relevant for a number of medical problems. These include iatrogenic rupture of fetal membranes due to perforation for amniocentesis or fetal surgery^1^, episiotomy and perineal tear during childbirth^2^, the tearing of skin, tendons and ligaments at lesions^3,4^ and the failure of sutures in soft tissues, e.g., with regard to the fixation of tissue-engineered vascular grafts^5^, skin grafts^6^ or heart valve leaflets^7^. The mechanical behavior of soft collagenous tissues (SCT) is complex, and correspondingly, the understanding and the prediction of failure at their defects is elusive, due also to the difficulties associated with the variabilities observed in corresponding mechanical experiments. Soft biological tissues are generally considered as defect tolerant, as eloquently portrayed by J.E. Gordon in his classic textbook^8^, and different arguments were proposed to rationalize this property^3,9–11^. High fracture toughness is often the consequence of intrinsic or extrinsic mechanisms opposing crack growth^12–16^, and many processes that increase fracture toughness are associated with dissipation of energy through inelastic deformations of material regions adjacent to the crack^12,14,15,17^. Conversely, several investigations on the fracture behavior of single collagen fibrils pointed at a rather brittle behavior, with only little inelastic deformation before failure^18–21^. Another mechanism that might explain enhanced toughness is the activation of long-range deformation and energy dissipation processes ahead of the crack [e.g., refs. ^22,23^]. The size of the material region in which these processes occur was suggested to be closely related to the critical size at which defects become influential, i.e., at which the material becomes flaw-sensitive^24^. Indeed, recent analyses of fracture experiments on dense connective tissue, cartilage and muscles, proposed the existence of large process and plastic zones ahead of the crack tip to explain why under certain circumstances these tissues are ignoring the presence of a crack^25,26^. On the contrary, our recent analysis of the nearfield of defects in fetal membranes indicated a markedly narrow zone affected by stress and strain concentration at the tip of macroscopic cracks, characterized by a strongly localized compaction of collagen fibers^27^.

In order to resolve these contradictions and to rationalize the fracture behavior of SCT, this work analyzes the deformation mechanisms in their crack nearfield and the conditions leading to crack propagation. To this end, we performed parametric studies using a novel hybrid computational approach combining continuum and discrete fiber network representations of the tissue. These in-silico investigations led to the formulation of specific hypotheses, which we then verified experimentally on SCT model systems, including fresh bovine Glisson’s capsule (GC) and a collagen type I based cell carrier material (CCC). Mechanical experiments were performed in-situ in a multiphoton microscope in order to characterize the collagen network response in the crack nearfield. While very effective at avoiding crack propagation, the highly localized processes observed to occur at the crack tip do not offer opportunities for extensive energy dissipation, and thus lead to a generally brittle behavior. SCT appear therefore to belong to a very peculiar class of materials, in that they are brittle and unable to activate long-range stress redistribution, but yet exhibit high defect tolerance. As a consequence, unique for this class, the very small size of the fracture process zone does not correlate with the extraordinarily large size of defects necessary to dominate the process of SCT failure. This work analyzes important implications of this peculiar behavior in the assessment and quantification of SCT fracture toughness for medical applications, such as suture retention strength, the reduction of tear resistance through defects and the dependence of fracture toughness on the osmolarity of the tissue environment.

## Results

### Size dependence of the tearing energy of SCT

**Fig. 1:**
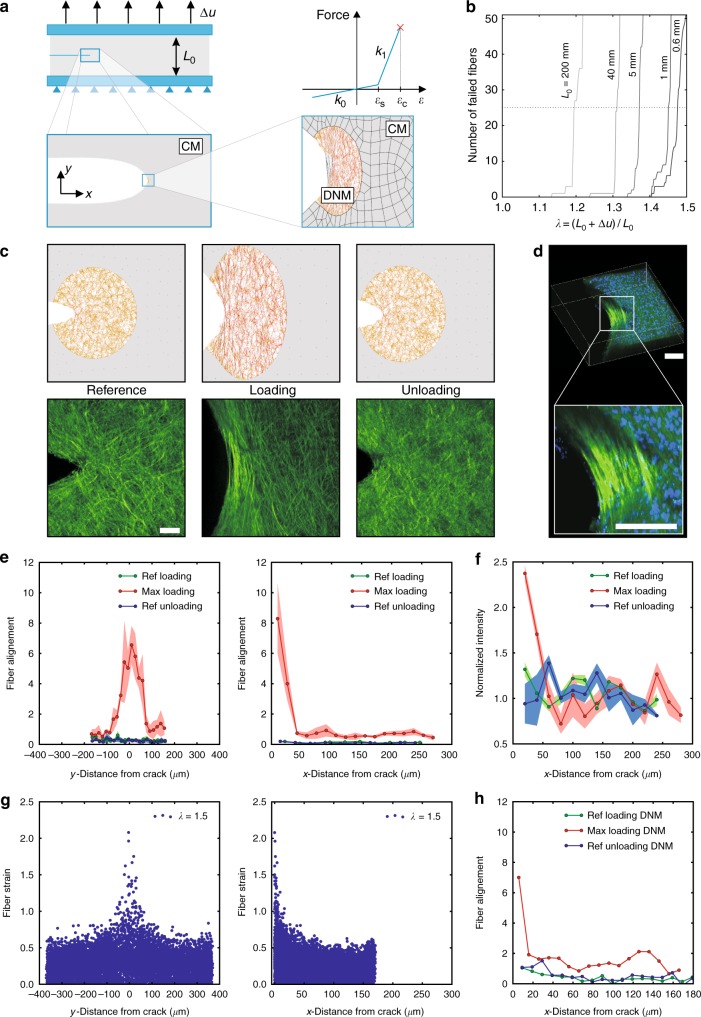
Near field analysis of notch-like defects. **a** Mode I fracture test configuration: a wide and short (initial length *L*_0_) testpiece with a preexisting notch is elongated up to failure. Soft collagenous tissue (SCT) simulations are performed with a hybrid approach, combining a continuum model (CM) in the far field of the defect and a discrete network model (DNM) in the near field. The fiber force law in the DNM is shown, with a small strain stiffness *k*_0_ and large strain stiffness *k*_1_ for *ε* > *ε*_s_, slackness strain, and limited by a critical fiber strain of *ε*_c_. **b** The global failure stretch *λ*_F_ depends on *L*_0_ as indicated by the fiber failure behavior. **c** Reversibility of fiber network deformation mechanisms: nearfield morphology in the reference state, (subcritical) loading and unloading for simulations and corresponding experiments in the multiphoton microscope (MPM, scale bar: 50 μm), for which the second harmonic generation signal (SHG) is shown. **d** 3D reconstruction of the defect with stained cell nuclei from MPM stacks in loaded condition (scale bar: 50 μm). **e** Degree of fiber alignment in MPM images at the notch along the direction of loading (*y*) and perpendicular to it (*x*), reported for reference, loaded and unloaded states. **f** Corresponding SHG signal intensity decay in the *x*-direction, normalized with respect to the intensity of the far field. **g** Fiber strain distribution from simulations in the nearfield along *x* and *y*-direction. **h** Fiber alignment is determined for three superimposed images of simulations, similar as in **e**. Results in (**e**, **f**) are shown as mean ± standard deviation

**Fig. 2:**
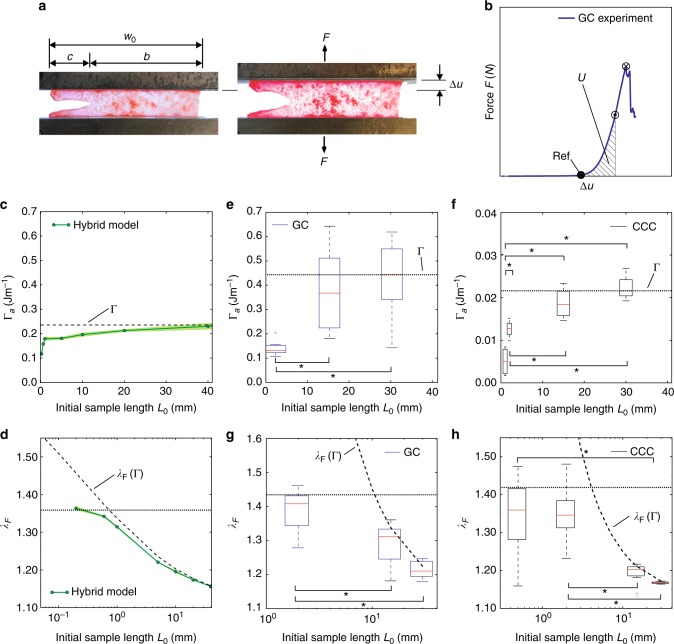
Apparent tearing energy depends on sample dimensions. **a** Images from mode I fracture experiments on Glisson’s capsule (GC) in reference and loaded state. **b** A representative force vs. relative displacement curve is shown for experiments on GC, indicating the reference state (filled circle), crack propagation (dot), catastrophic failure (cross) and the work until crack propagation *U*, which can be used to calculate the (apparent) tearing energy. **c**, **d** In-silico study (*n* = 3, in green, represented in mean and standard deviation) on the dependence of the apparent tearing energy Γ_a_ and the critical global elongation *λ*_F_ on the initial sample length *L*_0_. In **d**, the fracture mechanics prediction of *λ*_F_ based on Γ (dashed) and the critical elongation of intact samples (*n* = 3, dotted) are indicated. **e**–**h** Experiments on GC (*n* = 6, 9, and 5 for *L*_0_ = 2 mm, 15 mm and 30 mm, resp.) and CCC (*n* = 4, 4, 6, and 3 for *L*_0_ = 1 mm, 2 mm, 15 mm and 30 mm, resp.) in which Γ_a_ (**e**, **f**) and *λ*_F_ (**g**, **h**) are determined for different *L*_0_, reported as boxplots. Similarly as in **d**, fracture mechanics-based prediction of *λ*_F_ (based on mean Γ, dashed) and mean *λ*_F_ of intact samples (dotted) are shown in (**g**, **h**). In **e**–**h**, boxes represent upper and lower quartiles and lines inside the boxes define the median, while dots represent outliers, and whiskers 10–90 percentiles. Significant differences (*p* < 0.05) are indicated by * (Kruskal–Wallis)

The methodology introduced by Rivlin and Thomas^28^ to determine the tearing energy of rubber-like materials has been applied in previous studies for the characterization of SCT^25,26,29,30^. In the corresponding experimental configuration for mode I fracture testing (Fig. 1a) a short and wide specimen is elongated in the direction perpendicular to the notch, until crack propagation is observed. From the integral of the force-displacement curves, the corresponding value of the tearing energy can be calculated as the ratio *U*/*b* of the work of the external forces until crack propagation *U* and the effective sample width *b* (cf. Figs. 2a, b). We simulated such experiments using the hybrid model (see Methods), which combines a continuum representation of a SCT material in the far field and a discrete fiber network model for the near field of the defect (Fig. 1a). The fiber network model was recently shown to represent the deformation behavior of soft collagenous membranes, and in particular of GC, at macroscopic and microscopic length scales^31^. The single fibers were assumed elastic, and to break at a critical strain *ε*_c_ (Fig. 1a), in line with reported values of critical elongation of collagen fibers^18,21^. Simulations of mode I fracture tests indicate that the elongation *λ*_F_ at which the fibers break and the crack propagates, depends on the sample length *L*_0_ (Fig. 1b). For smaller samples, the critical elongation increases. Fracture mechanics theories explain this behavior based on the size dependence of the energy release rate associated with crack growth^28,32^. Remarkably, the hybrid model reproduces this result from a fiber level failure criterion, i.e., without the need to quantify the energy release rate associated with crack growth for a specific loading state. The second notable result of the simulations is that once a few fibers rupture, catastrophic sample failure follows (Fig. 1b), emulating the brittle behavior that was observed also in corresponding experiments with GC (Fig. 2b). Noteworthy, GC was chosen as a model system in the present investigations as it exhibits mechanical characteristics that are similar to those observed in other SCTs^31,33^, and it is available from the local abattoir in relatively large portions. The nearfield characteristics obtained from the simulations are compared with corresponding second harmonic generation (SHG) images from in-situ experiments on fresh bovine GC. These experiments were performed with specimens of 10 mm × 40 mm in free length and width, and a lateral cut of 10 mm. Both, the general morphology of the collagen fiber network and the localized fiber alignment observed upon mechanical loading are captured by the model (Fig. 1c). Indicative of the high reversibility of the deformation process, the nearfield fiber distribution reverts to its initial configuration after unloading from a subcritical loading state in both simulation and experiment (Fig. 1c, e, f, h). The higher SHG intensity at the notch tip clearly points at a highly localized response, with collagen network compaction in the direct vicinity of the tip (Fig. 1d). Noteworthy, low loading rates and dwell times were applied before imaging, thus reducing the presence of time-dependent effects (see Methods). At shorter time scales or higher loading rates, the outflow of water and, correspondingly, the compaction might be reduced. The detailed analysis of the images reveals fiber alignment (Fig. 1e) and intensity increase (Fig. 1f) ahead of the tip in GC within a region of approximately 50 μm width in the direction of the cut (*x*) and 100 μm height along the axis of elongation (*y*). At larger distances these values rapidly decrease to the corresponding levels that characterize the far field. These salient features of soft collagenous membranes observed in fracture testing are captured by the simulations based on the hybrid modeling approach: Fiber elongation and alignment peak at the notch tip and reduce to the corresponding far-field values at a distance of 40–80 μm and 100–200 μm in cut (*x*) and loading (*y*) direction, respectively (Fig. 1g, h).

The qualified model was used to investigate characteristic aspects of the fracture behavior of SCT, and to formulate hypotheses to be verified experimentally (Fig. 2a, b). At first, the dependence of the membrane tearing energy on sample dimensions was quantified by the computational model. The rupture condition in the model was defined as the loading state for which 25 fibers failed in the DNM. However, for a sufficiently large number of failed fibers, the results are practically independent of the failure criterion adopted (see Supplementary Discussion and Supplementary Fig. 2a). The calculated work of the external forces until crack propagation depends on the initial sample length. For this reason, the corresponding value of tearing energy is termed apparent tearing energy Γ_a_ in order to distinguish it from the material property Γ (tearing energy). Γ_a_ was computed for different values of *L*_0_ and converges to a stable value Γ_a_ = Γ only for large sample dimensions (*L*_0_ ≥ 40 mm), and therefore cannot be considered as a material property (Fig. 2c). Correspondingly, the fracture stretch *λ*_F_ computed for different sample lengths (Fig. 2d) shows that the critical elongation for *L*_0_ < 40 mm is lower than the one that would be predicted based on fracture mechanics, i.e., from the corresponding Γ (Fig. 2c). Note that results are shown to be nearly independent of the DNM model size and of the total number of considered fibers, for DNM radii > 100 μm (see Supplementary Fig. 2c, d). For sample lengths <1 mm, the critical elongation corresponds to the one calculated for a specimen without crack. Based on these computational findings, corresponding experiments were performed to validate the hypothesis that Γ_a_ converges to Γ only at large sample lengths for SCT. To this end, the fracture behavior of small (*L*_0_ of few mm) and large (*L*_0_ up to 30 mm) samples was analyzed (see Methods) for bovine GC (Fig. 2e) and, in addition, for CCC (Fig. 2f), which was selected as an example of a collagen-based material with a repeatable and well-controlled fabrication procedure, and is used as a stable and tough cell substrate^34,35^. The values of tearing energy measured with GC are not far from those predicted by the model (Fig. 2c, e). More importantly, these experimental results confirm the model predictions in that Γ_a_ increases with *L*_0_ and reaches a stable value only for larger specimens (*L*_0_ > 20 mm, Fig. 2e, f), and that the critical stretch deviates from the Γ-based predictions (Fig. 2g, h) for smaller samples. Previously reported Γ-values of SCT obtained in mode I fracture experiments^25,29,36–39^ used samples of lengths in the range of 4–30 mm. Thus, the results are likely to represent apparent values and to underestimate the real tearing energy of the materials. A similar conclusion was made in refs. ^25,26^ for muscle and cartilage based on an estimate of the critical distance^40,41^ for these tissues, which was larger than typical sample dimensions in fracture experiments^25^. This finding has been interpreted to be associated with large plastic and process zone dimensions in these tissues. Vice-versa, the present computational and experimental results demonstrate that in SCT the size of the crack near field is extremely small (Fig. 1e–h), which is a consequence of the collagen fiber densification mechanism at the crack tip (Fig. 1d). This process likewise can provide an effective shielding of local imperfections and might therefore be indicative of a general defect insensitivity of SCT. The influence of mm-sized flaws on the tear resistance of SCT was thus analyzed next.

### SCT are defect insensitive

**Fig. 3:**
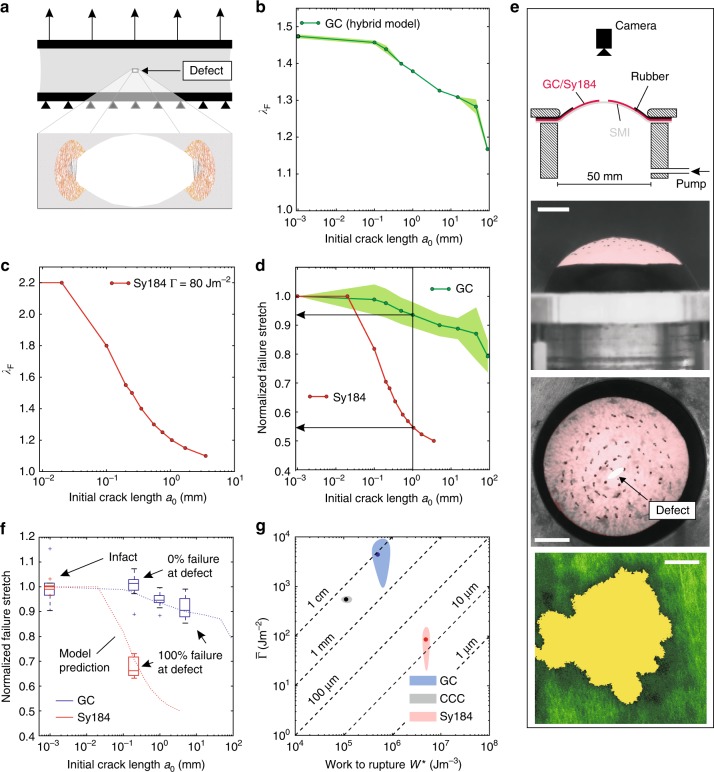
Defect tolerance of SCT and elastomers. **a** Biaxial tests of SCT containing a small defect: the simulated load case is illustrated, with the discrete fiber network model in the near field of the defect. **b** Results of the corresponding in-silico study for GC (*n* = 3), represented as mean and standard deviation. The computed global failure stretch *λ*_F_ is shown for different defect sizes. **c** The reduction of *λ*_F_ as a consequence of defects in an elastomer (Sy184) from fracture mechanics-based simulations using Γ = 80 J m^−2^ ^42^. **d** Sy184 (in red) and GC (in green) simulations are compared using the normalized *λ*_F_ with respect to the critical elongation of intact samples. **e** Experimental setup of the inflation experiments, where *λ*_F_ for defects of 0.2 mm, 1 mm and 5 mm (*n* = 10 for each defect size) is determined. Leakage is prevented by a layer of soft elastomer material. Representative top and side images for GC are shown and a 5 mm crack is indicated (scale bar: 10 mm). Black ink markers are visible on the tissue surface which were applied to facilitate measurement of deformations (see Methods). A MPM image documents the shape of a small defect with nominal length of 0.2 mm (defect in yellow, scale bar: 100 μm). **f** Experimental results for GC (in blue) and Sy184 (in red) for *λ*_F_ normalized with respect to the critical elongation in uniaxial tension (*n* = 5 each) experiments. The trendline from simulation (dotted red and blue) is indicated and experimental data are reported in boxplots, where boxes represent upper and lower quartiles and lines inside the boxes define the median, while dots represent outliers, and whiskers 10–90 percentiles. **g** Experimental results for the material tearing energy $\overline \Gamma$ (Sy184 from ref. ^42^) and the work to rupture W^*^^24^ in uniaxial tensile (*n* = 5 each) experiments, represented with mean and standard deviation

**Fig. 4:**
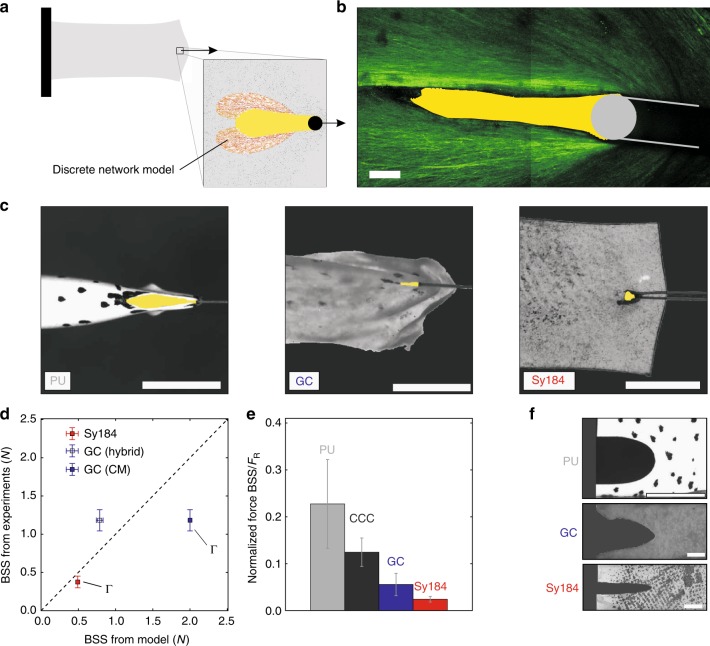
Analysis of suture retention strength. **a** Suture retention strength (SRS) test, illustrated based on the corresponding model for hybrid simulation, based on ref. ^29^. The defect is shown in yellow and the suture in black. **b** MPM image of a SRS experiment on GC, with SHG visualization of collagen fibers alignment. The shape of the defect is visible (in yellow, scale bar: 100 μm). **c** Images of suture retention strength experiments immediately before failure initiation for testpieces made of electrospun networks (polyurethane, PU), Glisson’s capsule (GC) and Sylgard184 (Sy184). Defects are highlighted in yellow, scale bar: 5 mm. **d** Pulling force on suture at failure initiation (BSS) as measured in SRS tests on GC (in blue, *n* = 5) and Sy184 (in red, *n* = 5). The experimental data are compared with model predictions based on fracture mechanics using Γ. The prediction based on the hybrid model approach is also reported. The dashed line corresponds to perfect agreement between experiments and computations. **e** BSS is normalized with respect to the critical force (*F*_R_) measured in uniaxial tensile tests. *n* = 5 SRS tests and *n* = 5 uniaxial tests were performed for each material. Results in (**d**, **e**) are presented as mean and standard deviation. **f** The shape of notches in mode I fracture experiments on PU, GC, and Sy184 are shown for a state close to crack propagation (scale bar: 5 mm)

The hybrid model was applied to predict the reduction in critical elongation *λ*_F_ associated with the presence of a defect in the center of a sample (Fig. 3a). The computational results, again based on a rupture criterion of 25 failed fibers, indicate a negligible reduction of the critical stretch for defects up to 0.1 mm, and a modest decrease for larger defect sizes up to a few mm (Fig. 3b). For comparison, a fracture mechanics-based analysis (see Methods) of the corresponding defect sensitivity of a soft silicon elastomer (Sylgard184 (1:10), Dow Corning, Midland, MI, USA) is also reported (Fig. 3c). The predicted reduction in failure stretch *λ*_F_ for a defect size of 1 mm is <10% for SCT and >40% for Sylgard184 (Fig. 3d). Based on these results, we prepared GC and elastomer samples containing cuts with lengths of 0.2, 1, and 5 mm, and loaded them in a biaxial membrane inflation experiment shown in Fig. 3e. In line with model predictions, all Sylgard184 samples failed at the defect, even for a defect size of 0.2 mm (Fig. 3f). Remarkably, the SCT ignored defects of 0.2 mm in that for none of the corresponding samples failure originated at the crack, while the failure stretch reduction for 1 and 5 mm was modest and much lower than for Sylgard184 (Fig. 3f). The reduction in critical stretch for SCT is thus in line with the predictions of the hybrid model and confirms the general defect tolerance of these materials. It was recently argued that the ratio between Γ and the work to rupture W^*^ of defect-free samples^24^ would provide a transition length scale at which flaws and defects begin to dominate the process of material failure. Corresponding plots (Fig. 3g) derived from data in Figs 2e–h, 4e and previous results^42^ indeed confirm that the estimated length scale match the simulations and experimental observations (Fig. 2d, g, h) in terms of order of magnitude, and indicate a 100-fold difference between GC and Sylgard 184. In fact, the length of flaw sensitivity of the SCT exceeds that of the vast majority of synthetic materials^24^.

Low notch sensitivity is associated with the ability of the collagen fiber network to generate a protective layer with high density around the defect, effectively preventing crack propagation. This enables a structure formed from a brittle material to be highly defect tolerant. Recent detailed microscopic analyses of amniotic membranes^43^ indicated the presence of micro-fractures in SCT with dimensions of up to 100 microns. Interestingly, our results indicate that the tissue might tolerate defects of such small dimensions without a reduction of its tear resistance. Larger cracks are generated in SCT as a consequence of needle perforation, e.g., for suturing in surgery. We therefore analyzed the ability of SCT to resist suture pull-out.

### Tearing energy does not predict suture retention strength

The suture retention strength (SRS) test is commonly used to quantify the force required to pull out the suture from the material^44^. Needle perforation generates a defect with dimensions in the mm range from which the failure process originates, and the question arises whether this type of failure can be captured by fracture mechanics concepts. SRS in-silico simulations (Fig. 4a) were performed using the hybrid approach with a discrete representation of the fiber network in the needle hole’s nearfield. Corresponding SRS tests were performed on different materials (Fig. 4c), and additionally in-situ for GC (Fig. 4b). The force for initiation of suture failure (referred to as BSS^29,45^) predicted by the model was compared with the values obtained from experiments on GC. The mean predicted value is about 30% lower than the measurements (Fig. 4d), in line with the lower fracture resistance previously predicted by the hybrid model for GC (cf. Fig. 2c, e). We then used a validated continuum model representation of GC^33^ and performed a fracture mechanics analysis^32^ of the suture test (see Methods), using the measured stable value of Γ of 0.45 J m^−1^ for GC (Fig. 2e). This provided a prediction for the suture failure force that is about a factor of two higher than the suture strength observed in the experiment (Fig. 4d). This means that the BSS of SCT would be largely overestimated if predictions were based on tearing energy values, i.e., the material property Γ, as would be the case in a fracture mechanics analysis. For SCT, Γ is thus relevant only for the analysis of large cracks (>10 or 20 mm), and this strongly limits its applicability for medical problems. For comparison, the same experiments and calculations were performed for Sylgard184, and for this material the computed energy release rate in suture pull-out tests agrees to a great extent with its tearing energy so that measured and predicted BSS values clearly match (Fig. 4d).

The BSS measured for the elastomer samples is much smaller than the value measured for GC, despite a similar strength of the two membranes. In order to quantitatively evaluate this superior ability of GC to resist the stress concentration associated with a suture, we normalized the BSS value with respect to the tensile force (*F*_R_) required to rupture an intact sample as measured in a conventional tensile test, using a sample with the same cross section dimensions as in the suture test. As expected, this ratio is by almost a factor of 2 larger for GC than for Sylgard184 (Fig. 4e). Even higher is the value obtained for CCC, another SCT specifically developed to provide high toughness^34,35^. We further hypothesized that a tissue consisting of fibers made of a material able to undergo significant plastic deformation would have enhanced defect tolerance through combining the advantages of the fiber compaction mechanisms in the near field with the ability to dissipate energy by inelastic deformation. Motivated by the known high fracture toughness of electrospun polymeric networks^9^, electrospun polyurethane (PU) mats were selected for verification of the hypothesis. Fracture tests indeed revealed very large plastic deformations in this material, effectively blunting the notch, providing local reinforcement and avoiding crack propagation (Fig. 4f). Notably, the normalized BSS measured for the PU mat exceeds the values for Sylgard184 and GC by factors of 10 and 4, respectively (Fig. 4e).

### Osmolarity of liquid environment affects fracture toughness

**Fig. 5:**
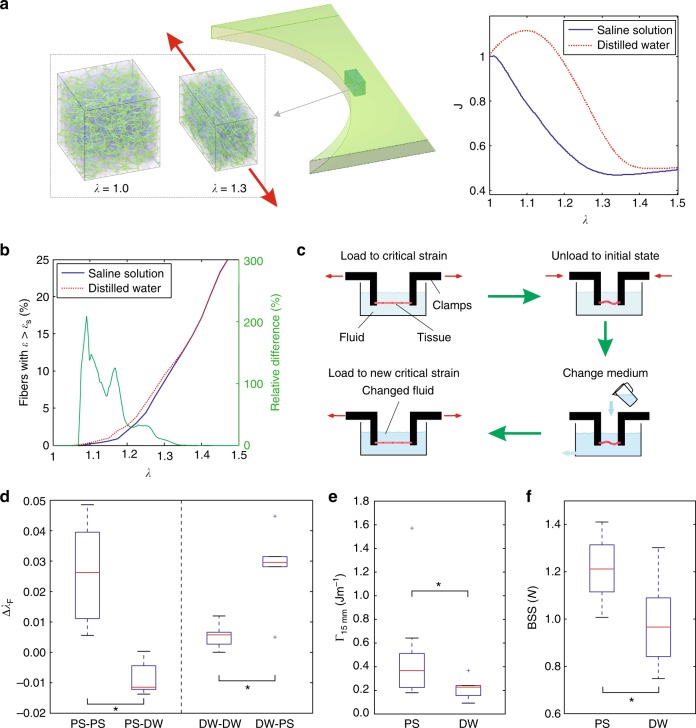
Osmolarity of the liquid environment influences the toughness of SCT. **a** Illustration of the 3D discrete fiber network model with volumetric chemoelastic contributions used to analyze the network deformation in the nearfield of the notch. Reference and elongated states are shown, together with the volume change *J* vs. *λ* curves. **b** The influence of bath osmolarity on the network kinematics at the notch tip is quantified in terms of the relative fraction of fibers with a strain larger than their slackness (5%). **c** Schematic illustration of the experimental procedure used for the investigation of the sample specific effect of bath osmolarity on fracture behavior of SCT. GC testpieces were immersed in physiological saline solution (PS) and in distilled water (DW), were elongated up to the point of crack propagation, then unloaded, after which the bath was either changed or not, before reloading to crack propagation. **d** Sample-specific difference in critical stretch Δ*λ*_F_ between subsequent loadings with (PS-DW, *n* = 5, and DW-PS, *n* = 5) or without (PS-PS, *n* = 6, and DW-DW, *n* = 7) bath change. **e** Experimentally determined tearing energy of GC in PS (*n* = 9) and DW (*n* = 5). **f** Suture retention strength tests on GC leading to smaller BSS in DW (*n* = 7) than in PS (*n* = 7). Results in (**d**–**f**) are presented as boxplots, where boxes represent upper and lower quartiles and lines inside the boxes define the median, while dots represent outliers, and whiskers 10–90 percentiles. Significant differences are indicated for *p* < 0.05 by * (Student *t*-test)

The compaction of fibers at the crack tip of SCT implies a strong volume reduction only possible through efflux of liquid from the network^27^. This essential ingredient of defect tolerance could therefore be affected by the chemical potential of the liquid environment of the tissue. In fact, lower osmolarity of the bath leads to increased water uptake by the tissue and thus higher hydrostatic pressure of the extracellular liquid. Accordingly, a higher magnitude of hydrostatic pressure is required to expel the water from the collagen network during tissue deformation^27^. This in turn is expected to translate into less fiber reorientation and therefore larger stretches for the collagen fibers^27^. In order to analyze these processes, a 3D fiber network model was created (see Methods) and boundary conditions representative of the state of deformation predicted with the hybrid model at the crack tip in the fracture test were applied (Fig. 5a). The 3D model contains a volumetric chemoelastic contribution accounting for the liquid phase of SCT. The chemoelastic behavior depends on the assumed concentration of fixed charges in the tissue and the density of mobile charges in the liquid environment, and the model parameters were selected to be representative of bovine GC. When subjected to elongation, calculations show that the tissue expels water and reduces its volume, as expected. Notably, if the surrounding environment is changed from 0.9% NaCl physiological saline solution (PS) to distilled water (DW), the model predicts that the amount of this volume reduction (dehydration) is lower (Fig. 5a). For uniaxial tension simulations in DW, volume even increases up to an elongation of 1.15, as the hydrostatic pressure of the liquid component is not sufficiently large to effectively expel water from the tissue. For larger global elongations, fiber alignment leads to hydrostatic pressure increase and thus volume decrease. Numerical results demonstrate that the amount of stretched fibers increases with elongation and, for the same global elongation, a larger amount of fibers are exposed to increased stretch in the DW case (Fig. 5b). This difference, however, reduces and finally vanishes for large elongations.

Fracture experiments in PS and DW were performed on GC samples (*L*_0_ = 15 mm) in order to quantify the effect of bath osmolarity on toughness. In order to test the sample-specific variation in critical elongation, experiments were performed with change of bath during the test, from PS to DW or vice versa. Samples were loaded till crack advance, unloaded and then re-loaded to reattain a level of elongation causing the next crack increment (Fig. 5c). The change in critical elongation was quantified for cases when the bath was changed between loading steps from PS to DW or vice versa, and compared to the case without change of bath (control). The results clearly demonstrate that the critical elongation is smaller when changing to DW, while it increases significantly for a change from DW to PS (Fig. 5d). Further evidence of the effect of the liquid environment on SCT fracture toughness is provided by a direct comparison of the tearing energy measured in mode I fracture tests in PS and DW, with significant differences between the two cases, despite the large scatter associated with these measurements (Fig. 5e). We also compared the suture retention strength measured in the two conditions and the results again indicate a reduction of the BSS in DW (Fig. 5f).

Hence, the experimental results clearly evidence a significant influence of bath osmolarity on toughness (Figs. 5d–f), whereas the model predicts that the bath has a notable effect on fiber kinematics only for moderate loads, while at higher, near-critical elongations the influence becomes small (Fig. 5b). Given the effect of environmental conditions on collagen fibril mechanics^20,46–48^, this discrepancy might point to a direct effect of bath osmolarity on the failure properties of the fibers themselves, in addition to the indirect effect resulting from the changed network kinematics.

## Discussion

We used a computational model of SCT able to account for the discrete nature and the non-affine deformation behavior of a collagen fiber network. In order to describe SCT fracture behavior, a simple failure criterion was introduced, assuming brittle failure of the fibers at a specific strain level. While there is evidence of generally brittle behavior of collagen fibrils in tensile experiments^18–21^, our model clearly represents a considerable simplification when compared with the complex molecular, intra- and interfibrillar damage mechanisms involved in collagen rupture in SCT^3^. Notwithstanding, this modeling assumption is supported by the sound agreement with experiments on GC and CCC. In particular, the morphology changes observed in the SHG crack tip images during loading-unloading cycles were fully recoverable, and all experiments indicated a generally brittle behavior of SCT, i.e., with catastrophic failure following after initial crack advance.

The model predicts important features of SCT fracture behavior, in particular the dependence of the apparent tearing energy on specimen dimensions and, perhaps more important, the defect insensitivity of SCT. This favorable characteristic is present despite the brittleness of the materials’ constituents and despite the strong localization of the deformation processes at the crack tip. As calculated with the model, and demonstrated with SHG images, at a distance of 100 microns from the crack tip, the state of deformation agrees with the far field. Thus, contrary to what is assumed in fracture mechanics theories^24,26^, for SCT the size of a defect to determine strength is by orders of magnitude larger than the size of the fracture process zone. Hence, we show here that materials exist, for which these two length scales are largely decoupled. To shed more light on the underlying mechanisms, we performed mode I fracture simulations with a model corresponding to a hypothetical network for which fibers display the same stiffness in tension and compression (Supplementary Discussion and Supplementary Fig. 4). In this case, the nearfield size is considerably larger and it becomes comparable to the characteristic sample length for transition to flaw insensitive response. Our results thus indicate that defect tolerance of SCT is neither associated with long-range deformation nor major dissipation mechanisms, but is a direct consequence of the highly non-linear fiber response, which facilitates their alignment and the strong lateral contraction, leading to a highly localized material densification at the crack tip. The resulting local reinforcement effectively shields the defect from increasing stress concentration and avoids premature failure. This observed defect tolerance is expected to be a characteristic of SCT with highly dispersed fiber distributions, such as skin, fetal membranes or pericardium. As a consequence of their flaw insensitivity (Fig. 3b, d, f, g), defects with size up to few mm are not expected to affect the tear resistance of SCT. Thus, under normal conditions, needle perforations or suture holes should not cause any measurable strength reduction. Extremely narrow nearfields in SCT further indicate that sutures placed at an inter-distance as low as a few mm are not expected to interact, and would hence not affect tissue integrity^49^. Experiments and simulations confirm that the estimated size of defects^24^ at which conventional fracture mechanics theories apply is in the range of several mm for SCT. Interestingly, this is two orders of magnitude larger than the size of their intrinsic flaws (up to 0.1 mm, see Supplementary Fig. 2l and ref. ^43^) indicating that the network is able to locally shield existing defects and thus avoid their propagation. While this mechanism provides SCT with high defect tolerance despite their brittleness, much-enhanced fracture toughness is obtained for materials combining the network compaction mechanism with large inelastic deformation and long-range inter-filament damage, such as electrospun PU mats. The demonstrated links between SCT fracture toughness, network compaction and the chemical potential of the SCT liquid phase points at relevant implications for mechanotransduction: The densification in the vicinity of a lesion creates a particular environment for resident cells, with strongly increased osmotic pressure of the extracellular liquid and enhanced stiffness of the extracellular matrix. The 3D model (Fig. 5a) predicts changes of osmotic pressure in the range of 10 kPa in the notch nearfield for a far-field elongation of 20% (Supplementary Fig. 1h), i.e., of a magnitude known to influence cell behavior^50,51^. These findings thus indicate that the peculiar deformation behavior of SCT in the near-field of cracks might not only serve as a protective mechanism to avoid their propagation, but, in addition, play a role in the mechanobiology of tissue repair and regeneration, e.g., through stimulating deposition and remodeling of the extracellular matrix.

## Methods

### Materials

Following an established protocol^33^, bovine Glisson’s capsule (GC) was separated from pieces of bovine livers, obtained from a local slaughterhouse, within 6 h after animal death. The collagen I based cell carrier CCC (Viscofan BioEngineering, Weinheim, Germany) was obtained from the manufacturer and immersed 24 h in physiological saline solution (PS, 0.9% NaCl) prior to testing. Electrospun polyurethane (PU) networks with an average fiber diameter around 900 nm were received from Empa (St. Gallen, Switzerland). PDMS (Sy184, Sylgard184, Dow Corning, Midland, MI, USA) samples were produced in a 1:10 mixing ratio of between crosslinker and base polymer^33,52^. GC and CCC samples were kept hydrated during sample preparation and testing in all experiments in PS or, if specified, in distilled water (DW).

### Sample preparation

For fracture tests, rectangular specimens were excised from GC and CCC, and a lateral cut of length *c* was introduced with a scalpel. The dimensions (i.e., the total length × width before clamping), free sample lengths *L*_0_ after clamping and *c* were 42 mm × 12 mm (*L*_0_ = 2 mm, *c* = 3 mm), 45 mm × 60 mm (*L*_0_ = 15 mm, *c* = 15 mm) and 60 mm × 60 mm (*L*_0_ = 30 mm, *c* = 15 mm). An additional set of experiments was performed with 40.5 mm × 6 mm CCC specimens (*L*_0_ = 0.7 mm to 1.1 mm, and *c* = 1 mm). Note that the total length is up to 4 times larger than *L*_0_ in order to provide sufficient clamping area. The adaption of *c* for each specific *L*_0_ was due to material availability considerations. For instance, with *c* = 15 mm for all samples, the required width for *L*_0_ = 2 mm would have been *w*_0_ = 60 mm, instead of the currently used 12 mm, thus leading to significantly larger testpieces and consequently a lower number of samples obtained from each liver. For uniaxial tension to failure tests (UA), dog bone specimens (ISO 37 Type 4, 2 mm width, *L*_0_ = 20 mm) were used. For inflation (EB) tests (GC, Sy184) circular samples with diameter 70 mm (free diameter *D*_0_ = 50 mm) were prepared, and central defects of dimensions of approximately 0.2 mm, 1 mm and 5 mm were created. For suture retention (SRS) tests^29^, 30 mm × 10 mm specimens were prepared (*L*_0_ = 20 mm), perforated and sutured by a needle and 5–0 monofilament suture (B. Braun Melsungen AG, Melsungen, Germany), with a bite depth of 2 mm away from the front edge. To allow for image-based analysis of in-plane deformation fields black markers were painted on specimen surfaces with a waterproof black pen (GeoCollege Pigment Liner 0.005). For in-situ testing in the multiphoton microscope, samples with free lengths of 10 mm × 40 mm (fracture, *c* = 10 mm) and 20 mm × 10 mm (SRS, bite depth 2 mm) were prepared and stained with DNA binding dye DAPI (40, 6-diamidino-2-phenylindole, dihydrochloride, Invitrogen) prior to testing to visualize cell nuclei using fluorescence.

### Experimental set-ups

The setup for uniaxial tension to failure (UA), SRS and mode I fracture testing^54–56^ combines hydraulic actuators with force sensors (MTS Systems, Eden Prairie, MN, USA, force range: 100 N) with a CCD camera system (Pike F-100B Allied Vision Technologies GmbH, Stadtroda, Germany) and 0.25× telecentric lens (NT55–349; Edmund Optics GmbH, Karlsruhe, Germany), allowing to simultaneously record top-view images, force (*F*) and clamping displacement (Δ*u*) signals (Figs 2a, b and 4c, f). For mode I fracture tests, custom 3D printed clampings with interlocking grooves were used^57^, and all clamps were equipped with sandpaper to reduce slippage and closed by screws. Mounting of the specimens was facilitated by sacrificial clamping jigs of sandpaper and plastic foil^31,33^. For GC and CCC clamps and testpieces were placed in an acrylic glass chamber and immersed in physiological saline solution (PS) at room temperature. Displacement and force signals were sampled at 10 Hz in UA and fracture tests, images were recorded at 2 Hz, and at 4 Hz in SRS tests. Equibiaxial membrane inflation (EB) tests were performed using a custom built setup^33,54,56^, where circular samples were clamped between the inflation cylinder and a cover ring (inner diameter *D*_0_ = 50 mm), fastened by screws, and loaded by pumping fluid into the cylinder. To prevent fluid leakage through the defect during inflation (Fig. 3e), a soft elastomer layer (SMI G/G 0.020”, Specialty Manufacturing Inc., Saginaw, MI, USA) was placed below the membranes. For GC, the device was placed inside an acrylic glass chamber filled with PS. The membranes were inflated by pumping additional PS into the aluminum cylinder with a syringe pump (Standard Infuse/Withdraw PHD Ultra Syringe Pumps, Harvard Apparatus, Holliston, MA, USA) controlled by custom LABVIEW (National Instruments, Huntsville, AL, USA) code, pressure was measured (digital manometer, LEX 1, Keller, Switzerland), an two CCD cameras (GRAS-14S5C-C, Point Grey, Richmond, BC, Canada) recorded top and side views at 1 Hz. In-situ SRS and fracture tests on GC (Figs 1c, d and 4b) were performed in a multiphoton microscope (MPM, Leica TCS SP8 Upright MP FLIM; Facility: Center for Microscopy and Image Analysis, University of Zurich) using a custom-built testing device with displacement and force sensors^27^, recording the second harmonic generation (SHG) of collagen and fluorescence stained nuclei, at 840 nm excitation wavelength.

### Fracture and SRS testing and analyses

Macroscopic mode I fracture tests on GC, CCC, and electrospun PU mats were performed with a strain rate of 0.3% s^−1^ (Figs 2a, b and 4f). Nominal tension *T* = *F*/*b* was calculated from the measured force *F* and initial ligament width *b*. Data analysis of samples is based on a small, specified tension threshold^33^ of 7 × 10^−4^ N mm^−1^, reached at a sample length *L*_r*ef*_. Nominal stretch in loading direction was thus *λ*_*N*_ = *l*/*L*_r*ef*_, where *l* denotes the current length *L*_r*ef*_ + Δ*u*. The local in-plane stretches *λ* (load direction) and *λ*_2_ (perpendicular to load direction) were obtained using a custom software^52^, which tracks features on the tissue surface in a specified region of acquired top-view images and reconstructs the homogenized in-plane deformation field. Initiation of crack propagation was identified by analyzing top view images, allowing the subsequent quantification of the corresponding failure stretch *λ*_F_ (Figs 2g, h and 5d) and force (Fig. 2a, b). *λ*_F_ is based on the measurements of local strains, thus unaffected by slippage at the clamps^42^. Following modifications^29,57^ of the classical analysis^28^ for soft tissues and thin materials, apparent membrane tearing energy (Figs 2e, f and 5e) was determined as ${\mathrm{\Gamma }}_{\mathrm{a}} = \gamma _{\mathrm{c}}L_0{\int}_1^{\lambda _{\mathrm{F}}} Td\lambda$, where *γ*_c_ is a factor accounting for the tissue slippage that is given through the ratio of nominal and local stretches at crack propagation^57^. Membrane tearing energy Γ was defined as the value of Γ_a_ measured for the largest samples. Note that the membrane tearing energy is ${\mathrm{\Gamma }} = \overline {\mathrm{\Gamma }} H_0$, where $\overline \Gamma$ is the characteristic energy of tearing and *H*_0_ the initial tissue thickness. The fracture mechanics-based predictions of *λ*_F_ were obtained by linear extrapolation of the mechanical response of the mode I fracture tests with *L*_0_ = 30 mm, and identifying the stretch necessary to reach Γ for various initial sample lengths (Fig. 2g, h). Membranes with a central defect were inflated by pumping 8 mL min^−1^ of liquid into the cylinder until rupture. Critical local in-plane stretches were identified from top-view images^52^ (Fig. 3e, f). UA tests were performed at a nominal strain rate of 0.3% s^−1^ until failure (cf. Supplementary Fig. 1a, b), the critical force *F*_R_, stretch *λ*_F_ and work to rupture W*  ^24^ was extracted from the force signal, clamping displacement and top-view images (Figs 2g, h, 3f, g and 4e). The tearing energy and W^*^ in Fig. 3g were calculated based on estimates for the initial sample thickness, where 100 μm was used for GC, 30 μm for CCC and 0.4 mm for Sy184. In order to compare critical force *F*_R_ with SRS tests (Fig. 4d), the critical tension was calculated and multiplied by SRS specimen width (10 mm) in order to get normalized forces for a similar cross-section in the two tests. For determination of SRS and break starting strength (BSS)^29^, the corresponding samples were clamped at one side, the suture loop was pulled slowly (0.2 mm s^−1^) until a threshold force (0.005 N) was reached, and then at 1 mm s^−1^ until failure occurred (Fig. 4a–c). From the force signal and analysis of top view images, the critical force BSS at crack propagation initiation was identified (Figs 4d, e and 5f).

### In-situ testing and analysis

Fracture and SRS tests were performed on GC in a multiphoton microscope. In-situ mode I fracture specimens were loaded stepwise at a rate of 0.1 mm s^−1^, and sutures were pulled at 0.1 mm s^−1^ in SRS tests. After each step and waiting time of ca. 2 min for stabilization of the force signal, 3D stacks in the region of the defect, i.e., the notch or suture hole, were acquired, with a step size of 1 μm along the membrane thickness (Figs 1c, d, 3e and 4b and Supplementary Fig. 2l). For the analysis of the spatial heterogeneity of fiber orientation, fiber alignment in loading direction (*y*) and perpendicular to it (*x*) was quantified based on principal component analysis^58^, and represented as the concentration parameter of the von-Mises distribution fit to the corresponding orientation histograms (Fig. 1e). This concentration parameter was represented in Fig. 1e and Fig. 1h to provide a dimensionless degree of fiber alignment. The analysis was applied independently to sub-images of 10 μm × 10 μm size in reference, loaded and unloaded state from five slices of each analyzed stack (Fig. 1e). Noteworthy, the same procedure was applied to rendered images of simulations (Fig. 1h) to determine the computed fiber orientations in an analogous way. Similarly, the intensity of the SHG signal was extracted from the sub-images as the average image brightness of 5 slices per stack (Fig. 1f).

### Computational material models

The 2D DNM^31^ was developed based on microscopical and histological data, representing the mechanical response of GC in uniaxial and biaxial experiments^31^. It consists of a cross-linked fiber network filled with continuum triangular finite elements that account for the resistance to fluid in- or efflux^27,59^. The model is generated by randomly placing cross-links at a density of *ρ*_c_ = 0.075 μm^−2^ within a specified domain^31^. Four connectors representing the fibers are defined for each cross-link based on a random weighted sampling process with uniform orientation distribution and a distribution in length resembling the shape of a Poisson distribution with mean *L*_c_ = 10 μm, if not specified otherwise. The wavy collagen fibers are modeled with axial connector elements (CONN2D2) in Abaqus (Abaqus 6.10-EF1, DS Simulia Corp., Providence RI, USA), and their non-linear mechanical response is approximated by a bilinear force-strain law *f*(*ε*) with a small stiffness *k*_0_ = 0.1 mN in the compression and initial uncrimping regimes, and *k*_1_ = 100 mN ≫ *k*_0_ when straight, i.e., *ε* > *ε*_s_, where *ε*_s_ is the slackness strain (Fig. 1a). Rupture of the fibers is considered when *ε* > *ε*_c_, setting fiber stiffness to zero (Fig. 1a). To numerically stabilize the model, a fifth connector of length *L*_c_ with zero elastic stiffness is added and each connector is equipped with a parallel linear dashpot element of small viscosity (*η* = 0.001 N s mm^−1^). Using the cross-links, a mesh of plane strain elements (CPE3) was generated by Delaunay triangulation with Matlab (Version 2015a, TheMathWorks Inc., Natick, MA, USA), and the elements were equipped with a volumetric hyperelastic constitutive law, defined by the strain-energy function^31,60^ Ψ = *C*(*J*^2^ − *J*^−*α*^)^2^, where *C* = 3.0 × 10^−8^ MPa and *α* = 6 are model parameters and *J* denotes area change. The selected model parameters lead to a low resistance to volume change down to a value of *J* = 0.4, i.e., close to the compaction limit of the network (Supplementary Fig. 1d). The influence of fiber slackness was studied varying *ε*_s_ ∈ {0.21, 0.04} (Supplementary Discussion and Supplementary Fig. 2). The effect of critical fiber strains was investigated by selecting *ε*_c_ ∈ {0.35, 0.6} (Supplementary Fig. 2), motivated by experimentally reported failure strains of collagen fibers. The DNM was either used to define representative area elements to compute homogeneous load cases or to define regions of hybrid models in which the DNM was combined with continuous material sections.

The hybrid models were established by coupling the DNM at the boundaries of the discretized domain with a continuum material that is representative of the DNMs homogenized macroscopic response (cf. Figs. 1a, c, 3a and 4a and Supplementary Fig. 1c, d).

The continuum model (CM) representative for SCT was based on a hyperelastic anisotropic constitutive model^33,61,62^ that represents *N* = 32 families of fibers uniformly distributed in the plane, with a slight off-plane inclination ±*ϑ*, and embedded in a weak compressible matrix. In the deformed configuration, these fibers are specified by the vectors **m**_*i*_, *i* = 1, 2, …, *N* and experience the fiber stretch $\lambda _i^{\mathrm{f}} = |{\bf{m}}_i|$. In terms of **m**_*i*_, the left Cauchy-Green tensor **b** and its determinant *J*^2^ = det **b**, the strain energy function reads Ψ = *μ*_0_(*e*^*qg*^ − 1)/(2*q*), with^33,62^

1 $$g = m_2({\kern 1pt} {\mathrm{tr}}{\kern 1pt} {\mathbf{b}} - 3) + \frac{{m_2}}{{m_5}}(J^{ - 2m_5} - 1) + \frac{{\bar m_3}}{{m_4}}\frac{1}{N}\mathop {\sum}\limits_{i = 1}^N \left\langle {\frac{{\lambda _i^{\mathrm{f}}}}{{\lambda _{\mathrm{s}}}} - 1} \right\rangle ^{2m_4},$$

where 〈…〉 denote Macaulay brackets and the material parameters were identified as *μ*_0_*H*_0_ = 2.15 N mm^−1^, *m*_2_ = 5.07 × 10^−3^, $\bar m_3 = 9.94$, *m*_4_ = 1.00001, *m*_5_ = 0.90, *q* = 6.15, *ϑ* = 5.77 × 10^−2^ [rad]. Different from previous versions^33,62^ the parameter *λ*_s_ was introduced to account for fiber slackness similar as in the DNM, and takes values of either 1.28 or 1.11. The Cauchy stress tensor **s** and Cauchy tension **T**_C_ = *λ*_3_*H*_0_**s** were calculated, where *H*_0_ is the initial thickness of GC, and *λ*_3_ denotes the stretch ratio along the membrane normal. The model parameters given above were identified by non-linear least squares minimization of the error between the responses of the CM and the parametrized DNM in simulations of UA, strip-biaxial (SB) and EB tests (cf. Supplementary Fig. 1c, d). The model was implemented as a user material in Abaqus, and used to describe the properties of the far-field regions, discretized by three- or four-node plane stress elements (CPS3, CPS4). Similar to the experimental procedure, a reference force was introduced for data analysis when post-processing the simulation results.

To study the osmotic environment on the three dimensional network kinematics of soft collageneous membranes a 3D hybrid chemoelastic model (cf. Figure 5, Supplementary Fig. 1h) was established coupling a 3D DNM^27^ with a mesh of tetrahedral finite elements that are equipped with chemoelastic material properties (Fig. 5). The network was generated by seeding cross-links at a density *ρ*_c_ = 5 × 10^−4^ μm^−3^ within the domain of a representative volume element (RVE) of the membrane. Fibers were then defined by four random connections between these crosslinks, uniformly distributed within the membrane plane and sampled from a distribution^27^ *p*(*l*, *φ*) leading to a von-Mises distributed out-of-plane angle *φ*, controlled by the concentration parameter *β* = 3, and Poisson-like distributed fiber lengths with mean *L*_C_ = 10 μm. The model was implemented in Abaqus software and fibers were discretized by connector elements (CONN3D2) with bilinear force-strain characteristics (*k*_0_ = 0.1 mN, *k*_1_ = 100 mN, *ε*_s_ = 0.01) analogous to the 2D DNM. Additionally, a tetrahedral mesh (C3D4) was generated through Delaunay triangulation of the crosslink, thus coupling this mesh to the non-affine motion of the network. The elements were furnished with chemoelastic properties^63^, given by a compressible neo-Hookean material and an additional osmotic pressure *π* resulting from Donnan’s equilibrium^64,65^, so that the Cauchy stress tensor reads

2 $${\bf{s}}_{{\mathrm{tetra}}}({\bf{b}},J) = \frac{{c\phi _{\mathrm{s}}^{{\mathrm{ref}}}}}{J}\left( {{\bf{b}} - J^{ - 2m}{\bf{I}}} \right) - \left[ {R\Theta \left( {\sqrt {c_{\mathrm{F}}(J)^2 + 4c_{{\mathrm{ext}}}^2} - 2c_{{\mathrm{ext}}}} \right) - \pi _0} \right]{\bf{I}},$$

where *c* = 8 kPa, *m* = 1.6 are material constants, *R* and Θ = 300 K stand for the universal gas constant and ambient temperature, and $\varphi _{\mathrm{s}}^{{\mathrm{ref}}} = 0.25$ is the solid volume fraction in the reference state. *π*_0_ is a constant that guarantees a stress free reference state^65^, and $c_{\mathrm{F}}(J) = c_{\mathrm{F}}^{{\mathrm{ref}}}(1 - \phi _{\mathrm{s}}^{{\mathrm{ref}}})/(J - \phi _{\mathrm{s}}^{{\mathrm{ref}}})$ the density of fixed charges in the tissue^66^, changing with volume from a reference value $c_{\mathrm{F}}^{{\mathrm{ref}}} = 20$ mol m^−3^ (cf. refs. ^66,67^). The salt concentration of the external bath was either *c*_ext_ = 154 mol m^−3^ for physiological saline or zero for distilled water. The homogenized RVE stress response to homogeneous boundary conditions was computed based on implicit FE analysis^68,69^, and the given model parameters match the response of the 2D DNM with a fiber slackness strain of 0.04.

Finally, the mechanical response of Sylgard 184 was modeled with an incompressible 4-term Ogden model^52,70^, with strain energy function ${\mathrm{\Psi }} = \mathop {\sum}\nolimits_{r = 1}^4 \mu _r\left( {\lambda ^{\alpha _r} + \lambda _2^{\alpha _r} + (\lambda \lambda _2)^{ - \alpha _r} - 3} \right)/\alpha _r$ and model parameters^53^*α*_1_ = 2.17, *α*_2_ = 9.06, *α*_3_ = 34.3, *α*_4_ = −5.40, *μ*_1_ = 0.291 MPa, *μ*_2_ = 0.00340 MPa, *μ*_3_ = 2.01 × 10^−11^ MPa and *μ*_4_ = −0.0115 MPa.

### Fracture and SRS analyses with the hybrid model

Mode I crack opening (Figs 1 and 2), fracture at defects in biaxial tension (Fig. 3) and SRS (Fig. 4) tests were simulated with the hybrid model in Abaqus. Finite element (FE) models of the SCT membrane samples were created with material properties given by the CM representative of GC. Defects were introduced by disconnecting FE nodes along lines representing the lateral or central cuts of defined length *a*_0_, and circular regions around the line ends were discretized by the 2D DNM. Calculations were performed for *ε*_s_ = 0.21 and *ε*_s_ = 0.04. Mode I fracture tests were simulated by prescribing the vertical (*y*) displacements (Δ*u*) of upper and lower edges of wide samples of aspect ratios *L*_0_:*w*_0_ = 1:6 and a lateral cut of length 0.25*w*_0_. Standard dimensions were 10 mm × 60 mm, and *L*_0_ was varied from 0.6 mm to 200 mm (Fig. 1a, b and Supplementary Fig. 1g). Samples were elongated by 50% of their length and subsequently unloaded. All simulations were repeated *n* = 3 times, if not specified otherwise, with networks realizations of the statistically defined DNM, and results are presented as mean ± standard deviation (Figs 1, 2 and Supplementary Figs 1 and 2). Moreover, the influence of various model parameters and failure criteria (i.e., number of failed fibers) on the results was studied (Supplementary Discussion and Supplementary Figs 2 and 4). The continuum mesh size was adapted with sample dimensions, while the cross-link density of the DNM was held constant in all simulations. The orientation and axial strain of the fibers were evaluated by post-processing the results at different stages of the simulation (Fig. 1a–c, g, h, Supplementary Discussion and Supplementary Figs 1e and 2b). Crack propagation (fracture) was defined by the failure of 25 fibers with failure strain *ε*_c_ = 0.35 or *ε*_c_ = 0.6 (see also Supplementary Discussion, Supplementary Fig. 2 and Fig. 1a, b). The corresponding *λ*_F_ = 1 + Δ*u*/*L*_0_ was identified (Fig. 2d and Supplementary Fig. 2a, d, f, h), and the total strain energy *U*(*λ*_F_) evaluated (Fig. 2b). The apparent membrane tearing energy Γ_a_ = *U*/*b* was then calculated by division with the ligament width *b* (Fig. 2a–c and Supplementary Fig. 2d, r, g, i). Critical fiber strain *ε*_c_ = 0.35 and slackness *ε*_s_ = 0.04 are used in computations in Fig. 2. In an analogous manner, the reduction in failure stretch *λ*_F_ in a SB specimen with a central defect of length *a*_0_ was investigated (Fig. 3a,b). *a*_0_ was varied between 30 μm and 90 mm, and sample dimensions were adapted accordingly such that *w*_0_ > 60*a*_0_ and *L*_0_ > 10*a*_0_ in all cases. *λ*_F_ was then normalized with respect to the ultimate stretch (Fig. 3d,f and Supplementary Fig. 2k), i.e., the elongation at which the defect-free (*a*_0_ = 0mm) sample ruptures. Computations in Fig. 3 are based on *ε*_c_ = 0.6 and *ε*_s_ = 0.21 for critical fiber strain and slackness, respectively, and the influence of failure criterion and various model parameters is addressed in the Supplementary Discussion and presented in Supplementary Fig. 2k. The same hybrid model was used for simulations of SRS testing. Computational models of rectangular samples (20 mm × 10 mm) were generated, furnished with GC properties, clamped on one edge and a triangular defect close to the other one was created. The suture cross-section was modeled as a circular rigid body of 100 μm diameter, in frictionless hard contact with GC, and with a suture bite depth of 2 mm from the lateral edge (Fig. 4a). Based on preceding studies circular domains with radius *r* = 150 μm were represented by the 2D DNM, in the regions where the maximal strains were expected, while the other regions were meshed by continuum elements (Fig. 4a). The displacement of the suture’s centroid was prescribed, the reaction force was analyzed and its value was identified as BSS when 25 connectors had failed on one side of the symmetric model (Fig. 4d).

### Fracture and SRS analyses with continuum models

All simulations with continuum models were performed in Abaqus. Nearfield and farfield deformation behavior in mode I fracture simulations with the CM (*λ*_*s*_ = 1.28) are compared with the hybrid approach, in terms of maximal principal in-plane stretches (CM) and peak fiber stretches (hybrid approach) along the loading direction and perpendicular to it (Supplementary Fig. 1e). Similarly, the CM for GC was compared with continuum formulations for Sy184 (Supplementary Discussion and Supplementary Fig. 1f). Fracture mechanics approaches were used to compute *λ*_F_ and BSS for the CM for GC (*λ*_s_ = 1.28) and the Ogden model for Sy184. The strain energy function *W* of the two models was used to compute *λ*_F_ from the implicit equation^28^ Γ = *W*(*λ*_F_)*L*_0_*H*_0_ (Fig. 2c, d). To determine *λ*_F_ in the presence of central defects in Sy184, crack opening simulation^32,71^ were performed to determine the energy release rate. Opposite nodes on a line extending from a defect of length *a*_0_ were tied together, the sample was stretched to a given *λ*, and the connection was released such that the crack increased in length by Δ*a*. From the corresponding change in energy Δ*U* we calculated −Δ*U*/Δ*a* for various initial lengths and values of *λ*, and the stretches *λ*_F_ and crack sizes corresponding to Γ = 80 J m^−2^ ^42^ were identified (Fig. 3c) and normalized by the experimentally determined ultimate stretch of Sy184 of 2.2 (Fig. 3d, f). Similarly, for SRS tests on GC and Sy184, the suture was pulled for a predefined displacement, followed by a release of tied nodes. The reaction force was analyzed at the suture’s centroid (BSS), and the energy release rate −Δ*U*/Δ*a* was calculated accordingly. Suture displacement was varied in iterative computations to obtain the experimentally determined values of Γ for GC and Sy184, and the corresponding BSS values were extracted (Fig. 4d).

### Statistical analysis

Data were analyzed, fitted and tested for statistical significance using Python or Matlab. Values in the present study are expressed as mean ± standard deviation or as boxplots. The significance between two different groups was analyzed with a two-tailed Student *t*-test. More than two groups were analyzed with Kruskal–Wallis and subsequent Dunn’s post hoc test. *p-*values of less than 0.05 were considered significant.

## Supplementary information

Supplementary Information

## Data Availability

All relevant data are available from the authors upon request, and/or are included within the main part and Supplementary Information.
