# Publisher Correction: Tear resistance of soft collagenous tissues

**DOI:** 10.1038/s41467-019-10560-y

**Published:** 2019-05-30

**Authors:** Kevin Bircher, Manuel Zündel, Marco Pensalfini, Alexander E. Ehret, Edoardo Mazza

**Affiliations:** 1 ETH Zurich, Institute for Mechanical Systems, 8092 Zurich, Switzerland; 2 Empa, Swiss Federal Laboratories for Materials Science and Technology, 8600 Dubendorf, Switzerland

**Keywords:** Biomaterials, Biomedical engineering, Bioinspired materials, Tissues

Correction to: *Nature Communications* 10.1038/ss41467-019-08723-y, published online 15 February 2019.

The original version of this Article contained errors in the third and fourth sentences of the ‘Computational material models’ section of the “Methods”, which incorrectly read ‘The model is generated by randomly placing cross-links at a density of *ρ*_c_ = 0.075 m^−2^ within a specified domain^31^. Four connectors representing the fibers are defined for each cross-link based on a random-weighted sampling process with uniform orientation distribution and a distribution in length resembling the shape of a Poisson distribution with mean *L*_c_ = 10 m, if not specified otherwise.’ The correct version states ‘*ρ*_c_ = 0.075 μm^−2^’ in place of ‘*ρ*_c_ = 0.075 m^−2^’ and ‘*L*_c_ = 10 μm’ rather than ‘*L*_c_ = 10 m’.

The second and third sentences of the fourth paragraph of the same section originally incorrectly read ‘The network was generated by seeding cross-links at a density *ρ*_c_ = 5 × 10^−4^ m^−3^ within the domain of a representative volume element (RVE) of the membrane. Fibers were then defined by four random connections between these crosslinks, uniformly distributed within the membrane plane and sampled from a distribution^27^ *p*(*l*, *φ*) leading to a von-Mises distributed out-of-plane angle *φ*, controlled by the concentration parameter *β* = 3, and Poisson-like distributed fiber lengths with mean *L*_c_ = 10 m.’ The correct version states ‘*ρ*_c_ = 5 × 10^−4^ μm^−3^’ instead of ‘*ρ*_c_ = 5 × 10^−4^ m^−3^’, and ‘*L*_c_ = 10 μm’ instead of ‘*L*_c_ = 10 m’.

This has been corrected in both the PDF and HTML versions of the Article.

