# Supplementary material for: Tear resistance of soft collagenous tissues
Source: Nat Commun. 2019 Feb 15;10:792. doi: 10.1038/s41467-019-08723-y (PMC6377632; doi:10.1038/s41467-019-08723-y)
Supplement: Supplementary file 1 — Supplementary Information [file 41467_2019_8723_MOESM1_ESM.pdf]

Supplementary Information

Tear Resistance of Soft Collagenous Tissues

Bircher et al.

Supplementary Discussion

Analysis of nearfield and farfield deformation behavior

Analysis of the deformation behavior in testpieces containing a crack requires characterization of the response to multiaxial (farfield) and uniaxial (nearfield) tension states. Uniaxial tension vs. stretch curves as well as the lateral contraction measured for CCC (Supplementary Fig. 1a) and bovine Glisson's capsule (GC, Supplementary Fig. 1b) reveal similar characteristics in the mechanical response, with larger stiffness and contraction values for GC. The mechanical behavior of the discrete fiber network model (DNM) is compared with a previously validated continuum model[1] (CM) of GC. The correspondence of the two models in the macroscopic multiaxial behavior in uniaxial (UA), strip-biaxial (SB) and equibiaxial (EB) tension states is shown over a large deformation range (Supplementary Figs. 1c,d). Results shown in Supplementary Figs. 1c-g are based on the parameters reported in the Methods, using a fiber slackness of ε_s=0.21 in the DNM, evaluated at a stretch of 1.4 (Supplementary Figs. 1c-f) or 1.2 (Supplementary Fig. 1g). Besides the macroscopic multiaxial response, the hybrid approach and the continuum model (CM) of GC are shown to provide very similar nearfield representations in mode I fracture simulations (Supplementary Fig. 1e), i.e. maximal fiber elongation and principal in-plane stretches are compared in the load direction (y) and in the perpendicular direction (x). The results show that the influence zone of the defect is generally small for the DNM, in the range of 100 µm in x and 400 µm in y direction, with a slightly larger nearfield size of the continuum model. The defect nearfield in continuum models representative of elastomers (using a Neo-Hooke and an Ogden model, see Methods) is significantly larger compared to GC in both x- and y-direction for the same applied elongation (λ=1.4, Supplementary Fig. 1f). The critical elongation increases for smaller sample initial length L_0 (cf. Fig. 1b), which is reflected in a corresponding effect on the notch shape: Supplementary Fig. 1g shows for a given nominal elongation considerably larger opening of the notch for larger values of L_0, leading to larger fiber strains at the tip (cf. Figs. 1b and 2d). The characteristic kinematics observed in the defect nearfield, i.e. fiber alignment and strong lateral contraction, correspond to that reported for SCT in uniaxial tension experiments[2–4]. In fact, the stress and strain states at the crack tip are very close to those of a uniaxial tension experiment. An analysis of corresponding fiber orientation histograms (cf. Fig. 1) was performed based on the so called collagen orientation index $\overline{COI}$, calculated based on a normal distribution fit of the orientation histograms, normalized with the corresponding value in the reference state[2,4–6]. A decrease of $\overline{COI}$ indicates an increase in fiber alignment in the loading direction. Average $\overline{COI}$ values of about 0.2 and 0.3 were obtained for experimental and computational results reported in Fig. 1, which are similar to the $\overline{COI}$ reported for under-critical loading in uniaxial tension experiments on human amnion and GC[2,4]. Supplementary Fig. 1h compares the osmotic pressure estimated based on the biphasic 3D network model (see Methods) for the notch nearfield and farfield in fracture tests. With increasing nominal elongation, network compaction leads to loss of fluid and thus osmotic pressure increase. This process is intensified at the notch, leading to a pronounced difference in mean osmotic pressure rise. Interestingly, also for moderate values of farfield elongation (i.e. λ=1.1) the model predicts a notable difference in mean osmotic pressure, in the range of few kPa. Along with network stiffening associated with nearfield compaction, osmotic pressure gradients are expected to contribute to the mechanobiological cues driving cells response and activating repair processes.

Analysis of failure behavior

Crack analysis based on a continuum model representation inevitably leads to a problem of singularity of deformation measures at the crack tip. This is obviated in fracture mechanics analysis through the quantification of energy release rate and J-integral[7–9], or through the introduction of a characteristic length scale for the assessment of the field quantities at a specific distance from the crack tip (e.g. ref[10]). Such workarounds are not needed for the hybrid modeling approach, which inherently incorporates a failure criterion and a characteristic distance associated with the network properties. General agreement with experimental observations supports the reliability of the model, which is thus qualified as a useful tool for investigation of factors (i.e. fiber network properties) potentially affecting the defect tolerance of connective tissue.

The influence of model parameters is investigated for the failure behavior in mode I fracture simulations (Supplementary Figs. 2a-i) and for the reduction of the critical elongation associated with the presence of a small central crack-like defect (Supplementary Fig. 2k). Calculations used the parameters presented in the Methods and the fiber critical strain and slackness were ε_c=0.35 and ε_s=0.21, if not specified otherwise, while the continuum model is adapted to match the deformation behavior of the network. Repeatability of the hybrid approach was investigated in terms of nominal failure stretch λ_F for different failure criteria, i.e. different number n_f of failed fibers (Supplementary Fig. 2a, n=3), as well as the decay of peak fiber strain in the DNM along the direction perpendicular to the loading (x) at the notch (Supplementary Fig. 2b, n=5). The small standard deviations indicate good repeatability of critical elongation and notch nearfield strain distribution (see also Fig. 2). Note that the choice of failure criterion (number of failed fibers n_f) has a significant impact on the magnitude of the critical stretch only for low n_f values, while results are independent of the failure criterion for $n_f \geqslant 25$. The size of the DNM region in hybrid approach simulations (Supplementary Fig. 2c) affects the critical stretch λ_F and the tearing energy Γ only for DNM radii less than 100μm (Supplementary Fig. 2d). This justifies the selection of a DNM radius of 150μm in all simulations (if not specified otherwise). The influence of the mean fiber length L_c in the DNM on Γ and λ_F indicates a significant effect of this parameter, with a tougher response for larger L_c (Supplementary Figs. 2e,f). Note that the crosslink density was adapted with varying L_c so that the macroscopic mechanical response remains relatively unaffected. The relative reduction in critical stretch associated with the presence of a notch seems less sensitive to variations of L_c. A similar analysis was performed for the influence of fiber behavior, i.e. ε_c and ε_s (Supplementary Figs. 2g,h). Γ as well as λ_F of notched and intact samples change significantly when the critical fiber strain is increased for the same slackness. Likewise, for the same critical fiber strain ε_c=0.35, a reduction of slackness results in higher Γ and lower λ_F values. Interestingly, the notch sensitivity, observed as the difference between notched and un-notched samples, increases for larger differences between critical and slackness strain (Supplementary Fig. 2h).

All variations in the DNM parameters (L_c, ε_c and ε_s) as well as the failure criterion (i.e. number of failed fibers) were shown to influence the absolute values of Γ in mode I fracture simulations. On the other hand, the characteristic dependence of the apparent tearing energy on initial sample length L_0 is comparable (Supplementary Fig. 2i) with a pronounced reduction of Γ_a for lower values of L_0. Correspondingly, the reduction of critical elongation in samples with small central defects (normalized with respect to intact samples) is shown to be only modestly dependent of the parameter selection (Supplementary Fig. 2k). These results indicate that substantial weakening is expected in SCT for crack sizes in the range of mm, while typical defects in soft collagenous membranes have dimensions of hundreds of microns, as reported in ref[11] for human amnion and shown for a representative defect in a GC sample in Supplementary Fig. 2l.

The influence of the notch depth c on the critical elongation λ_F and the tearing energy for a specified sample geometry (cf. Methods and Figs. 1a and 2a) in mode I fracture tests is investigated in a corresponding set of computations (n=1 network realizations), by varying c from 0 mm up to 30 mm for

initial sample lengths L_0=5, 10 and 20 mm. The DNM radius was set to 150 µm, and fiber slackness and critical elongation were ε_s=0.21 and ε_c=0.35, in line with the results presented in Supplementary Figs. 1 and 2. Supplementary Fig. 3a shows that the critical elongation is constant for c>5 mm, while it increases for smaller values of c. Obviously, for c=0 mm the critical elongation of an intact sample is measured. Similarly, a constant value of Γ_a is computed for c>5 mm (Supplementary Fig. 3b) and values are larger for smaller notch sizes, in line with an increasing value of the critical elongation. The validity of the results obtained with c>10 mm in the present investigations is thus confirmed. In fact, for c<5 mm false values of λ_F and Γ_a would be measured, as the geometrical criteria indicated for a mode I fracture tests are not satisfied, cf. ref[12]. Nearfield size is determined at under-critical loading states, i.e. just before crack propagation, by splitting up the x-axis in intervals of 10 µm, calculating maximum fiber strains for each interval and characterizing the nearfield as the region where the maximum fiber strain of an interval is larger than 105% of the maximum fiber strain in the stabilized farfield. The results show that for a notch size c<5 mm both peak fiber strain as well as nearfield size increase, while for c>5 mm they are practically independent of notch size (Supplementary Fig. 3c). On the other hand, in line with the results reported in Fig. 1, the nearfield size depends on L_0, and, similarly to Γ_a, it increases with a larger initial sample length.

The fiber compaction mechanism at the notch tip is associated with the network kinematics in a uniaxial tension state, as resulting from the very compliant response of the fibers in compression (governed through the fiber model parameter k_0) and very stiff in tension (k_1). We hypothesized that the nearfield behavior of the network would change significantly if the fiber response would display the same stiffness in tension as in compression, i.e. if k_0=k_1. Corresponding hybrid model simulations of mode I fracture tests were performed, and included a continuum model with adapted parameters so to provide a mechanical response similar to that of the modified DNM. Results are reported in Supplementary Fig. 4. Comparison with Supplementary Fig. 1e and with Fig. 2d indicates that the nearfield size is about 4 times larger than in the reference case and that the characteristic sample length for transition to flaw insensitive response is significantly smaller. The linear fiber response leads to a nearfield size (0.4-0.8 mm) that is comparable to the critical flaw dimensions (~0.3 mm), as is commonly the case for rubber-like materials[13]. Interestingly, the corresponding ratio between Γ and the work to rupture W^* (see Fig. 3g) leads to a transition length scale of 300 µm, i.e. more than 1 order of magnitude smaller than for the SCT models and thus closer to that of Sylgard 184.

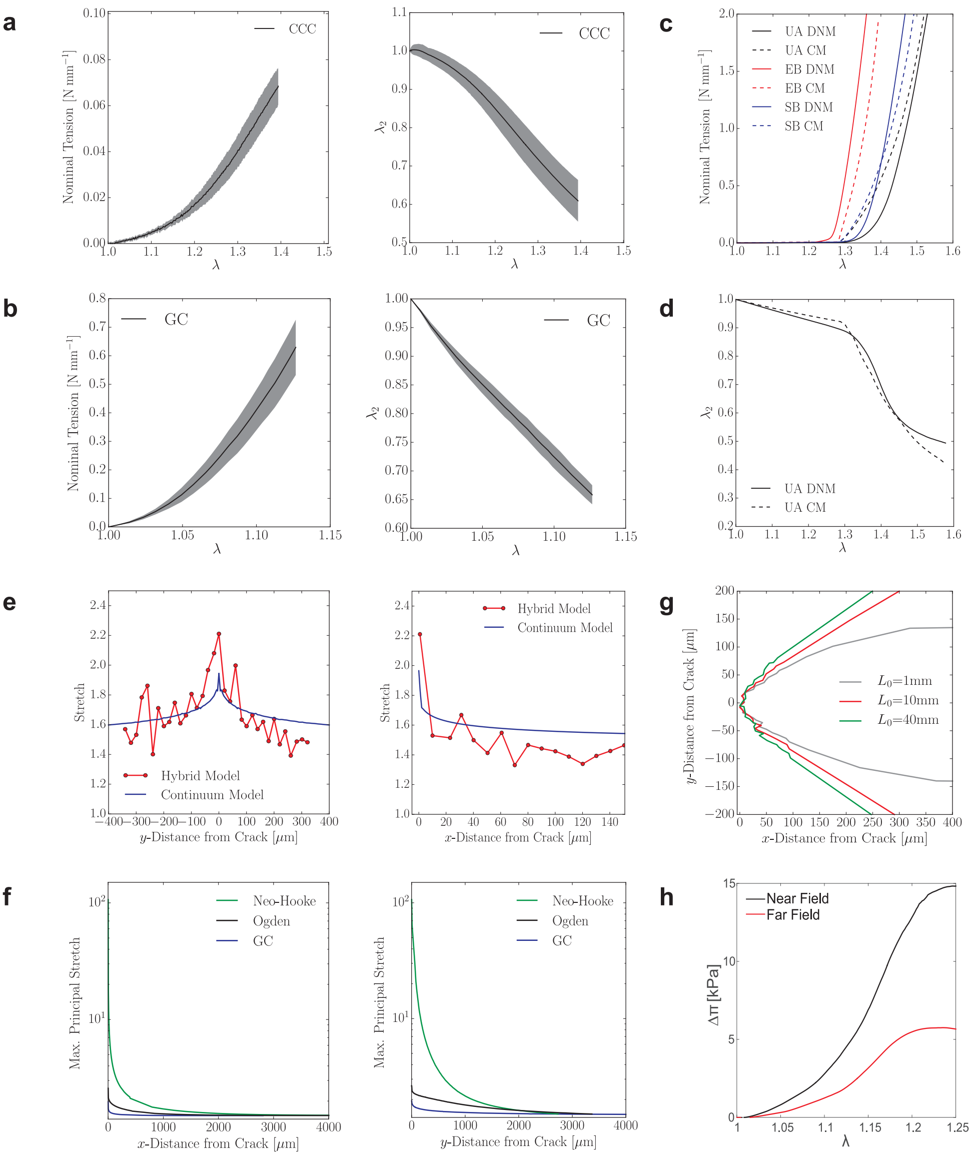

Supplementary Fig. 1 Analyses of tensile, nearfield and farfield behaviour. **a** Tension vs. stretch and kinematic response in uniaxial tensile (UA) experiments on CCC (n=5). Tension values are small due to the small membrane thickness (around 20 μm in dry state). Data are shown for a threshold force of 0.01 N and represented as mean and standard deviation. **b** Mechanical response of Glisson's capsule (GC) for n=5 UA experiments (threshold force 0.1 N). Data are represented as mean and standard deviation. **c,d**. Mechanical response for UA, SB and EB loading of the discrete network model (DNM, ε_s=0.21) compared with the corresponding continuum model (CM). Computations used the parameters from Methods, and are shown with respect to the initial configuration. **e** Peak fiber elongation (hybrid model) and maximal principal stretches (continuum model) in mode I fracture simulations. Decay is shown along the loading direction (y) and perpendicular to it (x) for a stretch λ of 1.4. **f** Maximal principal stretches decay in x- and y direction from the notch for continuum models representative of elastomers (Neo-Hooke and Ogden for Sylgard184) and GC evaluated at λ=1.4. **g** Notch shape predicted by the hybrid approach for different initial sample lengths (L_0=1 mm, 10 mm and 40 mm) at λ=1.2. **h** increase of osmotic pressure as computed with the biphasic 3D fiber network model at the notch tip (black) and in the farfield (red).

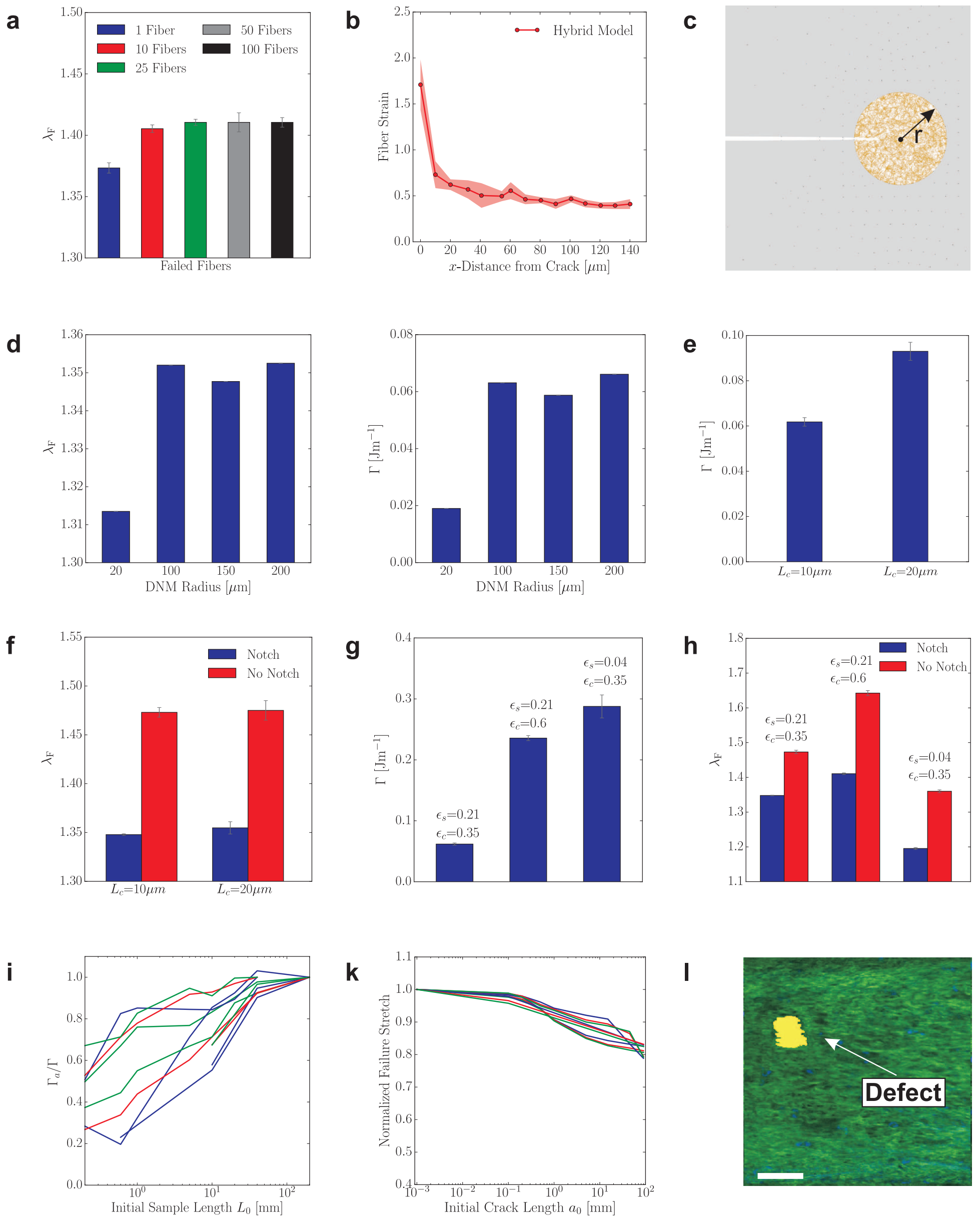

Supplementary Fig. 2 Influence of model parameters on the results of fracture computations. **a** The influence of the failure criterion (number of failed fibers) on the nominal failure stretch λ_F in mode I fracture simulations with the hybrid approach, shown for n=3 different DNM realizations. **b** Peak DNM fiber strain decay along the direction perpendicular to loading for n=5 realizations at a nominal stretch λ=1.5, without applying a fiber failure limit. Data are represented as mean and standard deviation. **c,d** Influence of the DNM region size (i.e. its radius "r", as indicated) on the critical elongation λ_F and the tearing energy Γ. Note that a new model was generated for every DNM size. **e,f** Tearing energy Γ and λ_F for mean DNM fiber lengths of L_c=10 μm and L_c=20 μm. λ_F in notched samples is compared to that of intact samples. **g,h** Effect of fiber slackness and critical strain on Γ and λ_F. For the latter, the results are compared with those for samples without a notch. **i,k** For all parameter variations (ε_s, ε_c, L_c and number of failed fibers) the evolution of Γ_a (normalized with respect to Γ) vs. initial sample length is shown (**i**). Likewise, for simulations of samples with small central defects (cf. Figure 3), the influence of model parameters on the reduction of the critical tissue elongation is quantified (**k**). **l** MPM image of GC in the reference state with a typical native defect. Collagen is shown in green from second harmonic generation, fluorescence stained nuclei in blue and the defect is colored in yellow. Scale bar: 100 μm. Results in (**a,e-h**) are presented as mean± standard deviation.

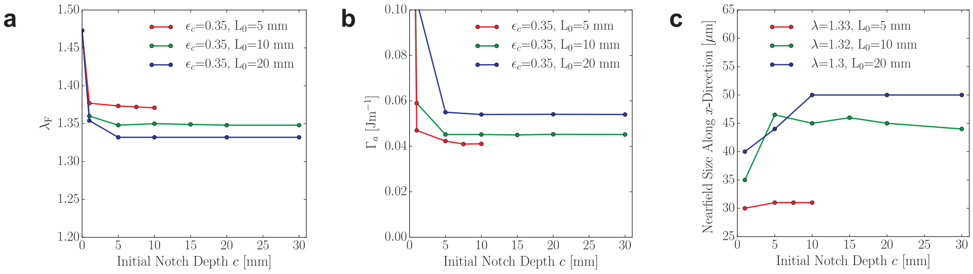

Supplementary Fig. 3 Influence of the notch depth in mode I fracture computations. **a** Critical elongation λ_F calculated for different notch depths c, for sample lengths L_0=5, 10 and 20 mm. 25 failed fibers were selected as failure criterion, for fibers with fiber slackness and critical elongation of ε_s=0.21 and ε_c=0.35. **b** Corresponding values of Γ_a. **c** For the corresponding under-critical loading states, the nearfield size along x-direction is determined. Note that the x-axis is split up in intervals of 10 μm and for each interval, the maximum fiber strain is determined. Then, the nearfield was defined as the region where the maximum fiber strains were larger than 105% of the maximum farfield fiber strain.

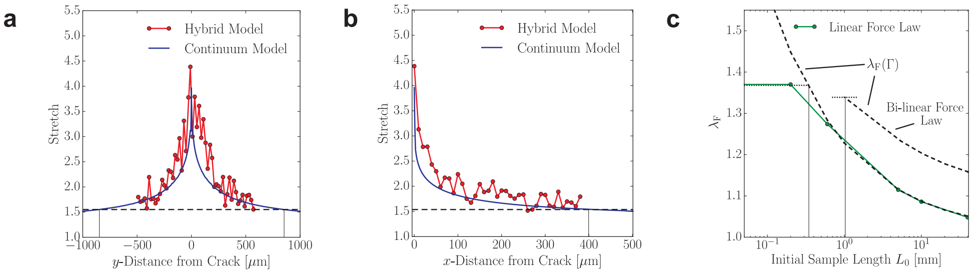

Supplementary Fig. 4 Influence of the fiber behavior in mode I fracture computations. **a,b** Peak fiber elongation (hybrid model) and maximum principal stretches (continuum model) in mode I fracture simulations for a linear force law of the fibers in the DNM and correspondingly adapted parameters of the continuum model. The decay is shown in in y-direction (**a**) and x-direction (**b**). These results are to be compared with those reported in Supplementary Fig. 1e. The dashed line represents 105% of the farfield stretch, and intersection with the continuum model indicates the nearfield size. **c** Dependence of λ_F on initial sample length L_0, for a failure criterion of 25 failed fibers (solid green), together with the fracture mechanics based prediction of $\lambda_F(\Gamma)$ (dashed) and λ_F of intact samples (dotted). Note that the fracture mechanics based prediction of $\lambda_F(\Gamma)$ and λ_F of intact samples of the reference model with the bi-linear force law is also shown (cf. Fig. 2d). For both models the transition length is indicated as the intersection of $\lambda_F(\Gamma)$ and λ_F of intact samples.

Supplementary References

1. Bircher, K., Ehret, A. E. & Mazza, E. Mechanical characteristics of bovine Glisson's capsule as a model tissue for soft collagenous membranes. *J. Biomech. Eng.* **138** (2016).

2. Bircher, K., Ehret, A. E. & Mazza, E. Microstructure based prediction of the deformation behavior of soft collagenous membranes. *Soft Matter* **13**, 5107–5116 (2017).

3. Ehret, A. E. et al. Inverse poroelasticity as a fundamental mechanism in biomechanics and mechanobiology. *Nat. Commun.* **8**, 1002 (2017).

4. Mauri, A. et al. Deformation mechanisms of human amnion: Quantitative studies based on second harmonic generation microscopy. *J. Biomech.* **48**, 1606–1613 (2015).

5. Feng, X. & Milanfar, P. Multiscale principal components analysis for image local orientation estimation. In *Conference Record of the Thirty-Sixth Asilomar Conference on Signals*, vol. 1, pp. 478–482. Systems and Computers (2002).

6. Mauri, A., Perrini, M., Ehret, A. E., De Focatiis, D. S. A. & Mazza, E. Time-dependent mechanical behavior of human amnion: Macroscopic and microscopic characterization. *Acta Biomater.* **11**, 314–323 (2015).

7. Lake, G. J. Fracture mechanics and its application to failure in rubber articles. *Rubber Chem. Technol.* **76**, 567–591 (2003).

8. Rice, J. R. A path independent integral and the approximate analysis of strain concentration by notches and cracks. *J Appl Mech-T ASME* **35**, 379–388 (1964).

9. Begley, J. A. & Landes, J. D. J integral as a fracture criterion. In *Fracture Toughness, Proceedings of the 1971 National Symposium on Fracture Mechanics, Part 11, ASTM STP 514*, pp. 1–20. American Society for Testing and Materials (1972).

10. Mazza, E. & Dual, J. Mechanical behavior of a μm-sized single crystal silicon structure with sharp notches. *J. Mech. Phys. Solids* **47**, 1795–1821 (1999).

11. Richardson, L. S. et al. Discovery and characterization of human amniochorionic membrane microfractures. *Am. J. Pathol.* **187**, 2821–2830 (2017).

12. Rivlin, R. S. & Thomas, A. G. Rupture of rubber. i. characteristic energy for tearing. *J. Polym. Sci., Part A: Polym. Chem.* **10**, 291–318 (1953).

13. Chen, C., Wang, Z. & Suo, Z. Flaw sensitivity of highly stretchable materials. *Extreme Mech. Lett.* **10**, 50–57 (2017).
